# Transcriptome Analysis of Tryptophan-Induced Resistance against Potato Common Scab

**DOI:** 10.3390/ijms23158420

**Published:** 2022-07-29

**Authors:** Pan Zhao, Lu Liu, Jingjing Cao, Zhiqin Wang, Yonglong Zhao, Naiqin Zhong

**Affiliations:** 1 State Key Laboratory of Plant Genomics, Institute of Microbiology, Chinese Academy of Sciences, Beijing 100101, China; liulu202206@163.com (L.L.); caojingjing@im.ac.cn (J.C.); wangzq1210@163.com (Z.W.); ylzhao97101@163.com (Y.Z.); 2 Engineering Laboratory for Advanced Microbial Technology of Agriculture, Chinese Academy of Sciences, Beijing 100101, China; 3 The Enterprise Key Laboratory of Advanced Technology for Potato Fertilizer and Pesticide, Hulunbuir 021000, China

**Keywords:** *Solanum tuberosum*, common scab, transcriptome analysis, tryptophan, induced resistance

## Abstract

Potato common scab (CS) is a worldwide soil-borne disease that severely reduces tuber quality and market value. We observed that foliar application of tryptophan (Trp) could induce resistance against CS. However, the mechanism of Trp as an inducer to trigger host immune responses is still unclear. To facilitate dissecting the molecular mechanisms, the transcriptome of foliar application of Trp and water (control, C) was compared under *Streptomyces scabies* (S) inoculation and uninoculation. Results showed that 4867 differentially expressed genes (DEGs) were identified under *S. scabies* uninoculation (C-vs-Trp) and 2069 DEGs were identified under *S. scabies* inoculation (S-vs-S+Trp). Gene ontology (GO) and Kyoto Encyclopedia of Genes and Genomes (KEGG) enrichment analyses indicated that Trp induced resistance related to the metabolic process, response to stimulus, and biological regulation. As phytohormone metabolic pathways related to inducing resistance, the expression patterns of candidate genes involved in salicylic acid (SA) and jasmonic acid/ethylene (JA/ET) pathways were analyzed using qRT-PCR. Their expression patterns showed that the systemic acquired resistance (SAR) and induced systemic resistance (ISR) pathways could be co-induced by Trp under *S. scabies* uninoculation. However, the SAR pathway was induced by Trp under *S. scabies* inoculation. This study will provide insights into Trp-induced resistance mechanisms of potato for controlling CS, and extend the application methods of Trp as a plant resistance inducer in a way that is cheap, safe, and environmentally friendly.

## 1. Introduction

The potato (*Solanum tuberosum*) is the fourth most important food crop in China after rice, wheat, and maize. In 2017, the potato-cultivated area was over 5.67 million hectares, and its production exceeded 99.15 million metric tons [1]. However, numerous diseases threaten the growth of potato, especially common scab (CS) disease [2]. CS in potato tubers results in skin lesions, leading to the production of unmarketable potatoes for fresh consumption, seeds and others [3,4,5]. In recent years, CS has caused a substantial economic loss worldwide and has become one of the most severe concerns for potato farmers [6,7,8]. 

CS is caused by the soil-borne Gram-positive, filamentous bacteria genus *Streptomyces* [9]. At least 12 species in *Streptomyces* have been reported to be able to produce scab symptoms [9,10,11], among which *S. scabies* is the first and best-characterized species [9,12,13]. Thaxtomins are plant phytotoxins secreted by *Streptomyces* and are supposed to induce CS skin lesion symptoms [3,9,14]. As the predominant form of thaxtomins produced by *S. scabies*, thaxtomin A inhibits plant cellulose biosynthesis, especially cellobiose and cellotriose biosynthesis [15,16,17]. The secretion of thaxtomin A in potato tubers alters plant physiologies, such as Ca^2+^, H^+^ inflowing, and polysaccharide deposition, resulting in tissue necrosis and reduced potato quality [10,18]. Genetic evidence has shown that eliminating critical genes of thaxtomin A synthesis, including *txtA*, *txtB* and *txtC*, negatively affects the pathogenicity of *S. scabies*. It is well-known that *txtA* and *txtB* encode two nonribosomal peptide synthetases, and *txtC* encodes cytochrome P450-type monooxygenase in thaxtomin A biosynthesis [9,19,20,21].

Although the causal agent of CS is well-diagnosed, it is still challenging to control CS effectively. The applications of individual strategies, such as crop rotation, chemical fumigation, or fungicides, have all proven insufficient [10,22]. Meanwhile, chemical methods are expensive and environmentally unfriendly. Therefore, new nontoxic and cheap strategies need to be searched for. Plant immunity inducers, which are green biological agents able to control diseases, have attracted much attention around the world. Immunity inducers are a class of immune-active compounds that can induce resistance in plants and promote healthy plant growth. Induced resistance in plants is triggered by biological or chemical inducers that protects the plant’s nonexposed parts against future attack by pathogenic microbes [23,24,25]. This resistance can be divided into induced systemic resistance (ISR), and systemic acquired resistance (SAR), induced by nonpathogenic microbes and pathogenic microbes, or based on the nature of the elicitor and the regulatory pathways involved [25]. SAR is dependent on the accumulation of phytohormone salicylic acid (SA) and pathogenesis-related (PR) proteins [26,27,28,29]. However, ISR is dependent of the phytohormone jasmonic acid/ethylene (JA/ET) [30,31]. Induced resistance is expressed not only locally at the site but also systemically in other parts of the plant that are separated from the inducer, enhancing the level of protection against a broad spectrum of attackers [32].

Plant-induced resistance provides a new strategy for controlling potato scab disease. In practice, foliar sprays of auxins and related molecules have been used for controlling CS [33,34,35]. Some chemicals act as elicitors or inducers that can trigger host immune responses [23,24]. For example, 2,4-dichlorophenoxyacetic acid (2,4-D), indole-3-acetic acid (IAA) [36], benzothiadiazole (BTH), β-aminobutyric acid (BABA), and acetylsalicylic acid (ASA) [33,34,35,37,38,39,40,41] have been used to suppress the development of CS. It has been reported that tryptophan (Trp), a precursor of auxin and secondary metabolites, could protect plants against some fungal, bacterial, and insect attacks [42,43,44,45,46]. Here, we found that the application of Trp could induce potato resistance to control CS. Considering that Trp is a relatively cheap chemical, this observation expanded the range of inducers that could be used to protect potato in the field. There are few reports stating that Trp application as an inducer can enhance the resistance of potato to CS. Thus, it is valuable to investigate the mechanisms of Trp-induced resistance against potato CS.

Transcriptome analysis is an important method for studying the expression of a large number of genes in a given tissue [47]. RNA transcript profiling can rapidly and effectively provide information for genome-wide transcript characterization, differential gene expression analysis, variant detection, and gene-specific expression. This technology is used to analyze the transcriptome in response to different biotic or abiotic stresses, including low-nitrogen stress [48], high-light stress [49], drought response to different soybean cultivars [50], salt stress [51,52], Cd stress [53], and sweet orange response to *Citrus tristeza virus* [54].

In this study, we analyzed transcriptome datasets from the leaves of potato plantlets. Leaves sprayed with Trp or water were examined from two groups (C-vs-Trp and S-vs-S+Trp) of potato plants. The differentially expressed genes (DEGs) of the two groups were identified to deepen the understanding of Trp-induced resistance to potato CS. These data will be critical to identifying defense-related genes that are regulated by exogenous Trp and to prompting our understanding of potato–*S. scabies* interactions.

## 2. Results

### 2.1. Foliar Treatment of Trp Enhances Potato Resistance against CS

Although Trp has been reported to protect plants against some fungi and bacteria, foliar application of Trp to defend against the soil disease CS has not been reported. It was hypothesized that Trp may act as an inducer to enhance resistance to CS in potato. Therefore, a field trial was designed to test the effectiveness of Trp. The results of disease incidence and yield indicated that 100 mg/L Trp was the best concentration (Figure S1).

**Figure 1:**
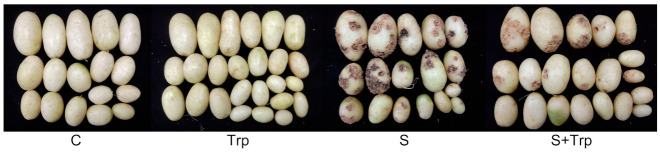
Disease symptoms of potatoes in pots. C: *S. scabies*-uninoculated plants treated with water; Trp: *S. scabies*-uninoculated plants treated with Trp; S: *S. scabies*-inoculated plants treated with water; S + Trp: *S. scabies*-inoculated plants treated with Trp.

Thus, 100 mg/L Trp was sprayed on potato plants in pots during tuber initiation. At this time, tubers are easily infected by the bacteria. The potato tubers were harvested from pots and assessed for CS. Tubers > 2 g were washed under running water and scored for disease incidence, disease index, total tuber mass and control efficacy. Tubers inoculated with *S. scabies* (S) showed strong susceptibility to disease compared to the control (C), showing that the inoculation was very successful. As shown in Figure 1, the scabs on the surface of the tubers treated with *S. scabies* and Trp (S + Trp) were reduced compared to tubers treated with only *S. scabies*. Then, we scored the total tuber mass, disease incidence, disease index and control efficacy. As shown in Table 1, the tuber mass of Trp was the best. After inoculation with *S. scabies*, foliar application of Trp decreased the disease incidence and disease index. Compared with S, the disease incidence of S+Trp was decreased from 90% to 59.09% and the disease index of S+Trp tubers was decreased from 53 to 27.27. The control efficacy of S+Trp compared with S was 48.54%. These results suggest that foliar treatment with Trp could enhance potato resistance against CS.

### 2.2. Analyses of the Transcriptome Datasets

The quality of the total RNA was assessed using the Agilent Bioanalyser 2100 (Agilent Technologies, Santa Clara, CA, USA) prior to subsequent experiments. A total of 12 RNA libraries were sequenced in depth, ranging from 102.19 to 105.84 million raw reads (Table S1). After filtering (removal of low-quality, joint contamination, and excessive unknown bases), 122.11 GB of data (fq.gz) was generated in total. More than 97.78% and 89.49% of the clean reads reached the Q20 and Q30 levels, respectively. More than 78.64% of the clean reads were mapped to the reference genome, including more than 58.61% unique reads (Table S2). The assessment of the filtered data indicated that the filtered sequences were high-quality.

**Figure 2:**
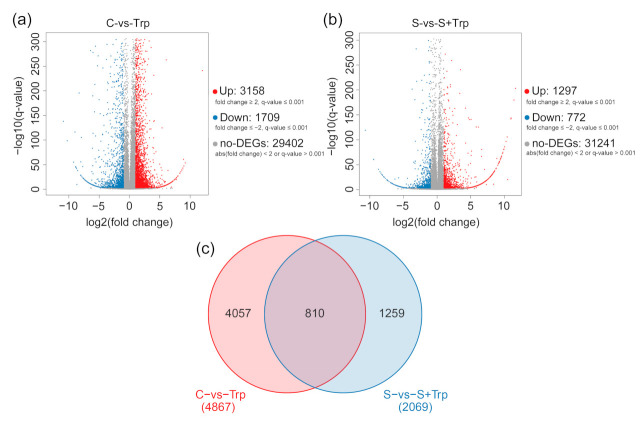
Volcano plot (**a**,**b**) and Venn diagram (**c**) of DEGs in the C-vs-Trp group and S-vs-S+Trp group.

The transcriptional levels were normalized using the fragments per kilobase per million reads (FPKM) method. Based on the criteria (|fold change, FC| ≥ 2 and Q-value ≤ 0.001), DEGs were defined. The number of DEGs was depicted as volcano plots (Figure 2a,b). Under *S. scabies* uninoculation (C-vs-Trp), compared with C plants, 3158 DEGs were upregulated and 1709 were downregulated (Figure 2a). Under *S. scabies* inoculation (S-vs-S+Trp), compared with the S-treated plants, we found that 1297 DEGs were upregulated and 772 were downregulated (Figure 2b). The C-vs-Trp group had more DEGs than the S-vs-S+Trp group. A Venn diagram analysis showed the unique and shared DEGs in the two groups (Figure 2c). In total, 810 DEGs were common between the two groups (Table S3). Among these 810 DEGs, 592 DEG showed the same trends in gene expression, including 387 DEGs being upregulated and 205 downregulated, and the remaining 218 DEGs had different expression trends between the two groups. These DEGs were involved in the starch and sucrose metabolism pathway, metabolic pathway, and plant hormone signal transduction pathway. There were 486 upregulated DEGs and 324 downregulated DEGs in the C-vs-Trp group. These genes were mainly categorized as catalytic activity and metabolic process, and were mainly involved in metabolic pathways, biosynthesis of secondary metabolites pathways and plant hormone signal biosynthesis. There were 506 upregulated and 304 downregulated DEGs in the S-vs-S+Trp group. Most of the genes were categorized as catalytic activity, metabolic process, transporter activity, and so on. The pathways or biological processes included biological regulation, binding, RNA transport, and cutin biosynthesis. Furthermore, 4057 DEGs, including 2627 upregulated DEGs and 1385 downregulated DEGs, were specific to the C-vs-Trp group (Table S3). These DEGs were involved in plant hormone signal transduction, plant-pathogen interaction, glycan degradation, and wax biosynthesis. 1259 DEGs, including 807 upregulated DEGs and 452 downregulated DEGs, were specific to the S-vs-S+Trp group (Table S3). These DEGs were involved in the mRNA surveillance pathway, base excision repair, and non-homologous end-joining pathway. These results showed that more DEGs were upregulated when treated with Trp without pathogens.

### 2.3. GO and KEGG Enrichment Analyses of DEGs

**Figure 3:**
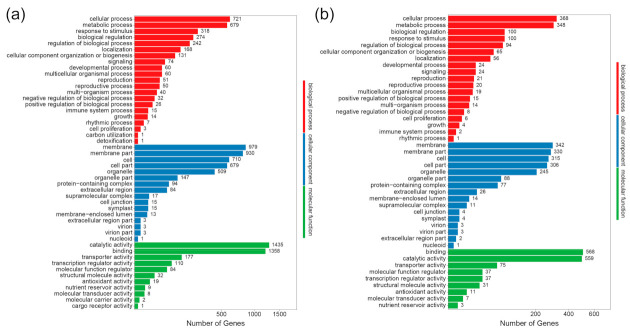
Gene Ontology (GO) enrichment analysis of DEGs in the C-vs-Trp (**a**) and S-vs-S+Trp (**b**) groups.

Gene Ontology (GO) enrichment analysis was applied to categorize the DEGs (Figure 3). In the two groups, DEGs were classified into three GO categories: biological process (BP), cellular component (CC), and molecular function (MF). In the C-vs-Trp group, the BP category was mainly enriched in cellular process (721 DEGs), metabolic process (679 DEGs), response to stimulus (318 DEGs), biological regulation (274 DEGs), and regulation of biological process (242 DEGs); in the CC category, DEGs were mainly enriched in membrane (979 DEGs), membrane part (930 DEGs), cell (710 DEGs), cell part (679 DEGs) and organelle (509 DEGs); in the MF category, the top five items were catalytic activity (1435 DEGs), binding (1358 DEGs), transporter activity (177 DEGs), transcription regulator activity (110 DEGs) and molecular function regulator (84 DEGs). In the S-vs-S+Trp group, the DEGs enriched were similar to those in the C-vs-Trp group. In the BP category, the top five items were cellular process (368 DEGs), metabolic process (348 DEGs), biological regulation (100 DEGs), response to stimulus (100 DEGs), and regulation of biological process (94 DEGs); in the CC category, the top five items were membrane (342 DEGs), membrane part (330 DEGs), cell (315 DEGs), cell part (306 DEGs) and organelle (245 DEGs); in the MF category, the top five items were binding (568 DEGs), catalytic activity (559 DEGs), transporter activity (75 DEGs), transcription regulator activity (37 DEGs) and molecular function regulator (37 DEGs). The results showed the same categorization of BP and MF in the two groups. It was also clearly visible that more genes were upregulated than downregulated for each group (Figure S2). There was a slight difference between the two groups. For example, in the “response to stimulus” process, there were more upregulated genes than downregulated ones in C-vs-Trp group. However, there were more downregulated genes than upregulated ones in S-vs-S+Trp group. The genes related hormones, stimulus, transcription regulator activity, and metabolic process should be given more attention in further research.

**Figure 4:**
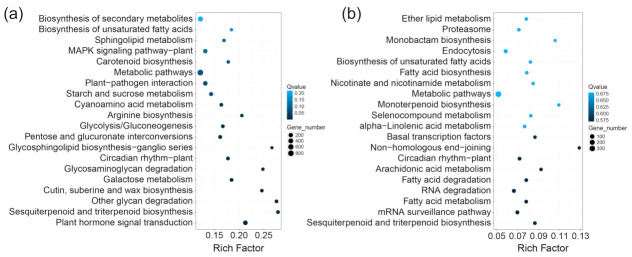
Kyoto Encyclopedia of Genes and Genomes (KEGG) pathway enrichment analysis of DEGs in the C-vs-Trp group (**a**) and S-vs-S+Trp (**b**) group. The X-axis represents the enrichment factor, and the Y-axis represents the pathway name. The depth of color represents the Q-value, and the size of the dot represents the number of DEGs.

Kyoto Encyclopedia of Genes and Genomes (KEGG) enrichment analysis was performed to further understand the DEGs. In the diagrams, the significant results were based on the Rich factor (RF) and Q-value (the smaller the Q-value is, the more significant the results; the larger RF is, the more significant the results). In the C-vs-Trp group, the major pathways were plant hormone signal transduction (268 DEGs, 0.21 RF), sesquiterpenoid and triterpenoid biosynthesis (55 DEGs, 0.28 RF), other glycan degradation (50 DEGs, 0.27 RF) and cutin, suberine and wax biosynthesis (38 DEGs, 0.25 RF); in the S-vs-S+Trp group, the major pathways were sesquiterpenoid and triterpenoid biosynthesis (17 DEGs, 0.09 RF), mRNA surveillance pathway (34 DEGs, 0.07 RF), fatty acid metabolism (19 DEGs, 0.08 RF) and RNA degradation (39 DEGs, 0.07 RF) (Figure 4).

### 2.4. Validation of RNA-seq Data by qRT–PCR

**Figure 5:**
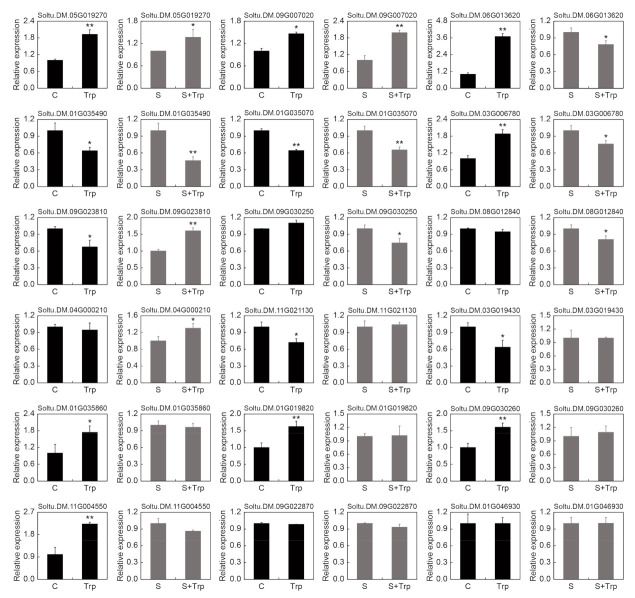
qRT–PCR validation of 18 DEGs. The asterisks denote statistically significant differences, as determined by Student’s *t*-test, * *p* < 0.05, ** *p* < 0.01. Three biological repetitions were performed.

To validate the transcriptome sequencing results, qRT–PCR was performed to assess the expression levels of genes. A total of 18 genes were selected, of which 7 were differentially expressed in the two groups, 3 were only differentially expressed in the S-vs-S+Trp group, 6 were only differentially expressed in the C-vs-Trp group, and 2 were not DEGs in both groups. As shown in Figure 5, the qRT–PCR results were consistent with the transcriptome sequencing results. The results indicated that the obtained RNA-seq data are reliable.

### 2.5. Candidate Trp-Induced DEGs in Phytohormone Pathways

The genes in the phytohormone pathways were correlated with induced resistance. Based on functional annotation and pathway analysis, several candidate DEGs related to phytohormone pathways were found to be notable in both groups. These were SA-, JA- and ET-related genes, such as SA biosynthesis-related genes *ICS* (*isochorismate synthase*), *PAL* (*phenylalanine ammonia-lyase*), SA-responsive genes *PR1* (*pathogenesis-related protein 1*) and *NPR1* (*nonexpressor of pathogenesis related genes 1*), NPR1 transcription factor gene *WRKY*, SA glucosylation genes *SAGT* (*salicylic acid glucosyltransferase*), JA biosynthesis-related genes *LOX* (*lipoxygenase*) and *OPR* (*12-oxophytodienoate reductase*), and ET biosynthesis-related genes *ACO* (*1-aminocyclopropane-1-carboxylate oxidase*) and *ACS* (*1-aminocyclopropane-1-carboxylate synthase*).

**Figure 6:**
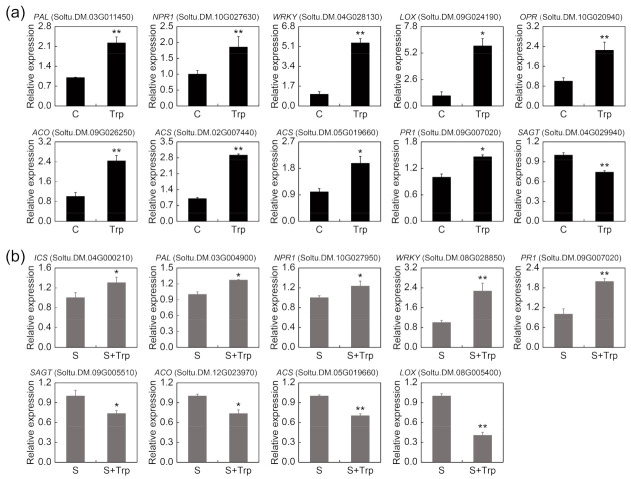
The relative expression of some candidate genes belonging to the C-vs-Trp group (**a**) and S-vs-S+Trp group (**b**) was detected by qRT–PCR. The asterisks denote statistically significant differences, as determined by Student’s *t*-test, * *p* < 0.05, ** *p* < 0.01. Three biological repetitions were performed.

In the C-vs-Trp group, the expression of SA biosynthesis-related genes *ICS* and *PAL* was significantly induced by Trp. All the *SAGT* genes were downregulated. Furthermore, three *NPR1* genes, one *PR1* gene and six *WRKY* genes were upregulated. Foliar treatment of Trp also induced the expression of JA biosynthesis-related genes. Seven *LOX* genes and two *OPR* genes were upregulated. The same results were seen in the ET-related genes. The expression of four *ACO* genes and seven *ACS* genes was induced (Table S4). Moreover, we selected some candidate genes with high FC values from the group and detected their relative expression levels by qRT–PCR (Figure 6a).

In the S-vs-S+Trp group, there were similar results in the expression of the SA biosynthesis-related genes. Two *ICS* genes and one *PAL* gene were upregulated. Three of the *SAGT* genes were downregulated. One *NPR1* gene, three *PR1* genes and two *WRKY* genes were upregulated. However, in the JA/ET biosynthesis-related genes, Trp downregulated genes expression. Four *LOX* genes were downregulated. The same results were seen in the ET pathway-related genes. The four *ACO* genes and one *ACS* gene were all downregulated (Table S4). Some genes with high FC values were selected to verify the differences in expression using qRT–PCR (Figure 6b).

## 3. Discussion

As a globally serious potato disease, CS is difficult to control via single management. Chemical methods are frequently used but expensive and environmentally unfriendly. The overuse or inappropriate use of chemical agents results in serious problems, especially environmental pollution and food safety, in agriculture [55,56]. Techniques to induce plant resistance represent a new and rapidly developing field of research and development [56]. To reduce the usage of chemical agents, we need to search for nontoxic and effective methods. Previous studies have reported that Trp could protect plants against some fungal, bacterial, and insect attacks [42,43,44,45,46]. In particular, Trp-derived secondary metabolites play important roles in defense responses, such as producing serotonin [57] and increasing the accumulation of camalexin, indole-carboxylic acid (ICA), and IAA [58,59]. Exogenous Trp can strongly inhibit the production of thaxtomin A by pathogenic *S. scabies* in a liquid thaxtomin-inducing growth medium [60,61]. Thus, the foliar application of Trp induces a broad-spectrum resistance exhibiting a great potential to avoid plant diseases.

We found that foliar treatment with Trp enhances potato resistance to CS and can increase its yield (Figure 1 and Figure S1). There are no reports that foliar application of Trp can control potato CS. Thus, it is of value to investigate the mechanisms of Trp-induced resistance against CS. We used transcriptome sequencing technology to analyze the mechanisms of the induced resistance of potato by foliar application of Trp against CS. A total of 4867 and 2069 DEGs were identified in the C-vs-Trp and S-vs-S+Trp groups, respectively. Based on GO and KEGG analyses, DEGs induced by Trp were found be involved in the metabolic process, response to stimulus and biological regulation (Figure 3 and Figure 4).

In view of the phytohormone pathways related to induced resistance, we analyzed key genes in hormone signaling pathways, such as SA, JA and ET. Induced resistance is an important mechanism by which plants enhance their defense ability via inducers in response to a broad range of pathogen attacks [30]. At present, the two forms of induced resistance, SAR and ISR, have been used in conventional agriculture against pathogens [62]. Via comparative transcriptome analysis, we found that SAR and ISR can be co-induced by Trp without pathogen treatment. Under *S. scabies* treatment, SAR is the pathway induced by Trp treatment.

SAR is required for the accumulation of PR proteins (and transcripts) and SA [26,27,28,29]. In this study, we analyzed the hormones of SA-related DEGs. In the C-vs-Trp and S-vs-S+Trp groups, the expression of SA-related genes was induced by Trp. *ICS* and *PAL* expression was upregulated, suggesting that SA biosynthesis may be increased. The *SAGT* genes were also downregulated. SAGT enzymes convert most of the produced SA to SAG, which is stored in the vacuole. Knockout mutants of the Arabidopsis *SAGT* genes showed increased disease resistance and free SA levels [63]. *AtSGT1* gene overexpression results in a reduction in SA content and reduced plant resistance [64]. SAR is typically characterized by augmentation of SA and *PR* genes’ activation. SAR-induced plants showed increased expression of SA-dependent *PR1* [29]. In our study, the expression of *PR1* genes in the two groups was upregulated by Trp. In fact, exogenous application of SA can activate *PR* gene expression and resistance in plants without pathogen inoculation [65,66]. These results imply that the SA signaling pathway is required to induce resistance by Trp.

ISR is mediated by a JA/ET-sensitive pathway and does not involve the accumulation of PR proteins or SA [30,31]. It is generally believed that this antagonism occurs between SA and JA, and we also analyzed the expression of SA. JA biosynthesis-related genes, including *LOX* and *OPR*, and ET synthesis-related genes, including *ACO* and *ACS*, were found. In the C-vs-Trp group, the expression of *LOX*, *OPR*, *ACO*, and *ACS* was shown to increase after treatment with Trp. Surprisingly, the expression of these responsive genes was downregulated by foliar treatment with Trp in the S-vs-S+Trp group. There were significant differences in the expression of these genes between the two groups. Both synergistic and antagonistic interactions between SA and JA have been reported [67]. The interaction between SA and JA is either concentration-dependent or tissue-specific and dynamic [68]. In our results, we presumed that Trp-mediated resistance is different with or without pathogen attack. Without pathogens, foliar treatment with Trp induced SA and JA increases at low levels. Therefore, the resistance induced by Trp overlaps with ISR and SAR. This was also found in a recent study showing that the SA- and JA-biosynthesis pathways can be co-induced [69]. In contrast, in the S-vs-S+Trp group, SA biosynthesis-related genes were induced, and JA-related genes were downregulated. This showed that SAR was induced by Trp rather than ISR when the pathogen was inoculated. *NPR1* expression is consistent with *ICS* and *PAL*, which are SA biosynthesis genes. NPR1 is a common regulator of ISR and SAR [66]. The SAR and ISR pathways are independent but have an overlapping requirement for NPR1 [70]. In both groups, the expression of *NPR1* was increased. These results showed that Trp-mediated resistance required NPR1 to regulate the SAR and ISR pathways in pathogen inoculation. This is different from Si-mediated LB resistance in potato that occurs through ET/JA- and NPR1- dependent signaling pathways [71]. In a study of SA- and JA-mediated gene expression, the WRKY70 transcription factor was likely to be involved in mediating SA-JA crosstalk [72]. However, in our data, no *WRKY70* genes were found among the DEGs. Whether the other transcription factors play a role is unclear; we require further analysis of our data in future works.

There are many elicitors that can initiate the plant defense response [62]. These include many chemical compounds, such as SA, BTH, BABA, or PBZ, or biological compounds including metabolites, oligosaccharides, glycoproteins, glycopeptides, proteins, polypeptides, lipids, and other cellular components [56]. Amino acids, such as methionine, can activate the ROS defense pathway and induce defense-related genes [73]. Lower concentrations of JA and BTH enable the simultaneous expression of both SAR and ISR pathways [74]. The combination of ISR and SAR can increase protection against pathogens that are resisted through both pathways in addition to extending protection to a broader spectrum of pathogens than ISR/SAR alone [29]. Trp-induced resistance is unlike these elicitors. In the S-vs-S+Trp/C-vs-Trp groups, there are different signaling pathways involved in Trp-induced resistance. Further research, such as protein assays, plant hormone measurements, and cell assays, is needed to validate the functions of interesting genes.

In this study, we revealed that foliar treatment with Trp can induce resistance of potato against CS. Our results showed that Trp induced resistance though different pathways under different conditions. Under pathogen inoculation, SAR is the pathway induced by Trp treatment. Without pathogens inoculation, SAR and ISR can be co-induced by Trp. This study provides useful information for research into Trp-induced resistance mechanisms and extends the application of Trp as an alternative agent to control CS, especially considering the field conditions.

## 4. Materials and Methods

### 4.1. Determine the Proper Concentration of Trp

To determine the proper concentration of Trp, a series of concentrations solutions (50 mg/L, 100 mg/L, 200 mg/L, and 400 mg/L) were prepared in water. The field trial was arranged in a plot in which CS occurred year by year. The plot consisted of five subplots of the same area (4.2 m × 4 m) that sowed the same number of seeds at the same time. The different concentrations of Trp were sprayed on field potato plants 3 weeks after seed germination. Water was sprayed as the control. Trp solution or water was sprayed onto the leaves of different potato groups until runoff. Field tubers were harvested at 120 days after seeding and assessed for disease incidence and yield.

### 4.2. Plant Materials

The CS-susceptible potato cultivar, Shepody, was used. The potato plantlets were multiplicated through plant tissue culture technology and grown in pot trials. Briefly, stems of potato plantlets were cut into node explants of similar length. These single-node explants were cultured on solid Murashige and Skoog (M&S) medium (pH 5.8) at 24 °C in an 8 h/16 h light cycle. Two weeks later, five established non-embryogenic callus lines of similar size were selected and transferred into pots filled with the same amount of autoclaved vermiculite. The selected potato lines were maintained under the same growth conditions. In brief, the pots were cultured at 24 °C with a 16 h light/8 h dark photoperiod and 70 µmol m^−2^ s^−1^ photon flux density provided by fluorescent lamps. For further trials, there were 3 plots (diameter 25 cm) that 5 seedlings per pot to replicate each treatment.

### 4.3. Pathogen Inoculation and Tryptophan Treatments

The *S. scabies* strain 4.1765, a highly pathogenic isolate from the China General Microbiological Culture Collection (CGMCC), was used in our experiments. The *S. scabies* strain was cultured on an oatmeal medium (OM) plate for 15 days at 28 °C. Then, the pathogenic colonies were inoculated into tryptic soy broth (TSB) and incubated at 28 °C in a rotary shaker (200 rpm) for three days. The cell pellets were harvested by centrifugation (10 min, 5000× *g*) and washed with sterile water. The bacterial cells were resuspended in fresh water to a final concentration of 1 × 10^9^ conidia/mL for inoculation. This concentration of the suspension was determined based on the relationship between amount of pathogenic *S. scabies* and the incidence of CS on tubers [75,76]. Then, 10 mL inoculation buffer was poured near the roots of seedlings. Therefore, a total of 50 mL inoculation buffer was inoculated into each pot.

The potato plants were divided into two groups and maintained under the same growth conditions. One was subjected to *S. scabies* infection, and another group was *S. scabies* uninoculation. Twenty-one days post-inoculation, at which time the tubers initiate and bacteria are highly infectious [77,78], Trp solution or water was sprayed onto the leaves of different potato groups until runoff. The concentration of the tryptophan solution was set at 100 mg/L, because this relatively low concentration could effectively inhibit the spread of CS disease. For the *S. scabies*-uninoculated plants, leaves were treated with tryptophan (Trp) and water (C). The same treatment of the leaves was performed in the *S. scabies*-inoculated plants (S+Trp and S).

After 4 days treatment, two top leaves were collected from each plant. Leaves collected from 5 different plants in one pot were pooled together as one sample for RNA extraction. Three replicates were repeated for each sample.

### 4.4. Disease Assessment

Tubers harvested 90 days after seedlings were planted in pots (3 pots, 5 plants per plot) were graded and assessed for CS. Tubers > 2 g were washed under running water and scored for the percentage of tuber area covered by scab.

The disease index was calculated by the following equation:

Disease index = [∑(n × 1 + n × 2 + n × 3 + n × 4 + n × 5)/(N × 5)] × 100. (n = number of tubers corresponding to the numerical grade. N = total number of potato tubers assessed. 5 = high score on the severity of scale). The percentage of tuber area covered: 0. No symptom of scab; 1. 0–12.5%; 2. 12.6–25%; 3. 26–50%; 4. 51–75%; 5. 76–100%.

Control efficacy = (disease index of control − disease index of treated)/disease index of control × 100%.

### 4.5. RNA Extraction and RNA Sequencing

In total, twelve samples of four conditions were used for total RNA extraction and library construction. Total RNA was isolated using the RNeasy Plant Mini Kit (Qiagen, Hilden, Germany) and library construction and sequencing were conducted by The Beijing Genomics Institute (BGI, Beijing, China, http://www.genomics.cn/index.html; accessed on 6 May 2022). The BGISEQ-500 platform was used for RNA sequencing and generated raw data [79,80]. The raw reads of the transcriptome data were filtered with SOAPnuke software to remove unsatisfactory reads with low quality, joint contamination, and excessive unknown bases.

The clean RNA-Seq data have been deposited in the SRA database under NCBI (Accession No. PRJNA611872).

### 4.6. Data Mapping, Analysis and Functional Annotation

The filtered clean reads were saved as FASTQ data and aligned to the potato reference genome *S. tuberosum* group phureja DM1-3 v6.1 (http://spuddb.uga.edu/; accessed on 20 April 2022) by HISAT [81,82,83].

The updated reference genome was generated by adding the newly identified transcripts into the original potato reference sequences. The clean reads were mapped to the updated reference sequences by Bowtie 2 [82,83,84]. The abundance of transcripts was estimated using the FPKM [85] method.

DEGs were analyzed by DEGseq [86,87] with the parameters “FC ≥ 2” and “Q-value ≤ 0.001”. GO functional enrichment of DEGs was performed based on the GO database (http://www.geneontology.org/; accessed on 22 April 2022), and pathway enrichment of DEGs was performed based on the KEGG database (http://www.genome.jp/kegg/; accessed on 23 April 2022). Meanwhile, GO term enrichment and KEGG pathway enrichment analyses were conducted using the “phyper” function of the R package. FDR < 0.05 was defined as the threshold for significant enrichment.

### 4.7. qRT–PCR Analysis

Total RNA was extracted using the Plant Total RNA Extraction kit (Tiangen, Beijing, China) according to the manufacturer’s instructions. cDNA was synthesized from 1 µg total RNA using the TransScript^®^ One-Step gDNA Removal and cDNA Synthesis SuperMix (Transgene, Beijing, China). qRT*–*PCR was performed using the SYBR^®^ Green Realtime PCR Master Mix (Toyobo, Osaka, Japan). All qRT*–*PCRs were analyzed using CFX96 Touch (Bio-Rad, Hercules, CA, USA) with three technical replicates and three biological replicates. The relative expression levels of selected genes were identified using the 2^−ΔΔCT^ method [88]. *StActin* (Soltu.DM.04G007480) was used as an internal control. The primers for qRT*–*PCR are shown in Table S5.

## Figures and Tables

**Table 1:** Disease index and incidence assessment following foliar application of Trp on CS.

Treatment	Total Tuber Mass (g)	Disease Incidence (%)	Disease Index	Control Efficacy (%)
C	323.40 ± 7.82	0	0	–
Trp	370.51 ± 1.70	0	0	–
S	271.13 ± 5.85	90.00 ± 7.08	53.00 ± 6.95	–
S + Trp	342.67 ± 10.89	59.09 ± 2.53 **	27.27 ± 1.18 **	48.54 ± 8.27

The asterisks denote statistically significant differences, as determined by Student’s *t*-test, ** *p* < 0.01. Three biological repetitions were performed.

## Data Availability

The clean data presented in this study are available in the Sequence Read Archive (SRA) database in NCBI (Accession No. PRJNA611872).

## Supplementary Materials

The following supporting information can be downloaded at: https://www.mdpi.com/article/10.3390/ijms23158420/s1.
