# Supplementary figures and images for: Transcriptome Analysis of Tryptophan-Induced Resistance against Potato Common Scab
Source: Int J Mol Sci. 2022 Jul 29;23(15):8420. doi: 10.3390/ijms23158420 (PMC9369096; doi:10.3390/ijms23158420)

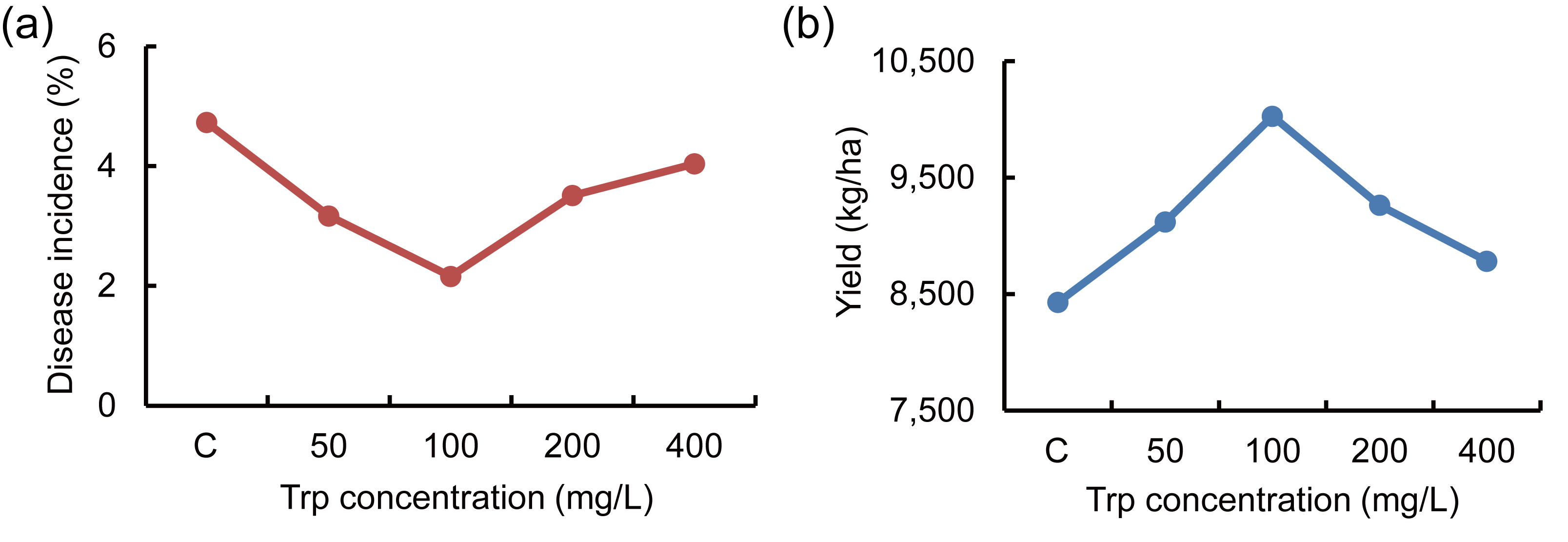

Supplement: Supplementary file 1 [file ijms-23-08420-s001.zip › Figure S1. Disease incidence (a) and yield assessment (b) following foliar application of Trp on in filed.tif]
